# Validation of two scales for measuring participation and perceived stigma in Chinese community-based rehabilitation programs

**DOI:** 10.1186/s12955-018-0938-3

**Published:** 2018-05-29

**Authors:** Eva Yin-han Chung, Gigi Lam

**Affiliations:** 1 Department of Special Education and Counseling, The Education University of Hong Kong, 10 Lo Ping Road, Tai Po, New Territories Hong Kong; 2 School of Medical and Health Sciences, Tung Wah College, 31 Wylie Road, Homantin, Kowloon Hong Kong; 3 School of Arts and Humanities, Tung Wah College, 31 Wylie Road, Homantin, Kowloon Hong Kong

**Keywords:** Participation, Disability, Stigma, Community-based rehabilitation

## Abstract

**Background:**

The World Health Organization has asserted the importance of enhancing participation of people with disabilities within the International Classification of Functioning, Disability and Health framework. Participation is regarded as a vital outcome in community-based rehabilitation. The actualization of the right to participate is limited by social stigma and discrimination. To date, there is no validated instrument for use in Chinese communities to measure participation restriction or self-perceived stigma. This study aimed to translate and validate the Participation Scale and the Explanatory Model Interview Catalogue (EMIC) Stigma Scale for use in Chinese communities with people with physical disabilities.

**Methods:**

The Chinese versions of the Participation Scale and the EMIC stigma scale were administered to 264 adults with physical disabilities. The two scales were examined separately. The reliability analysis was studied in conjunction with the construct validity. Reliability analysis was conducted to assess the internal consistency and item-total correlation. Exploratory factor analysis was conducted to investigate the latent patterns of relationships among variables. A Rasch model analysis was conducted to test the dimensionality, internal validity, item hierarchy, and scoring category structure of the two scales.

**Results:**

Both the Participation Scale and the EMIC stigma scale were confirmed to have good internal consistency and high item-total correlation. Exploratory factor analysis revealed the factor structure of the two scales, which demonstrated the fitting of a pattern of variables within the studied construct. The Participation Scale was found to be multidimensional, whereas the EMIC stigma scale was confirmed to be unidimensional. The item hierarchies of the Participation Scale and the EMIC stigma scale were discussed and were regarded as compatible with the cultural characteristics of Chinese communities.

**Conclusion:**

The Chinese versions of the Participation Scale and the EMIC stigma scale were thoroughly tested in this study to demonstrate their robustness and feasibility in measuring the participation restriction and perceived stigma of people with physical disabilities in Chinese communities. This is crucial as it provides valid measurements to enable comprehensive understanding and assessment of the participation and stigma among people with physical disabilities in Chinese communities.

## Background

Community-based rehabilitation (CBR) aims to promote the rights and opportunities for people with disabilities [39]. Through CBR programs, people with disabilities are enabled to participate in their community and society. Within a human rights framework, CBR is promoted to remove the obstacles, barriers, and discrimination that hinder the participation of people with disabilities. It is also advocated to promote the active participation of people with disabilities and their caregivers through appropriate measures to attain their maximum independence and full participation in all aspects of life [24]. Participation refers to involvement in life situations [40]. Problems an individual may experience in involvement in life situations are classified as participation restrictions [9]. Activity limitations and restrictions on participation are more critical to the affected person than the underlying health condition. Evidence on the social participation of people with disabilities is essential in program planning, monitoring, and assessing the effect of interventions aimed at reducing participation restrictions. Knowledge regarding the degree of participation restriction of a person is useful in informing the progress of the person as a result of an intervention. However, there is no universal accepted definition of participation [16], participation restrictions are a very widespread phenomenon, and scientific evidence and data on participation restrictions are limited [36].

Social stigma and discrimination constitute a critical environmental factor that limits participation and contributes to disabilities [37]. Stigma is regarded as a set of prejudices, stereotypes, discriminatory beliefs, and biases linked to the characteristics that differentiate a person from others [15]. Social stigma is defined as the attitudes of others toward people with disabilities; enacted stigma refers to the actual episodes of discrimination against people with disabilities; felt stigma is the stigmatization as experienced by the person; and self-perceived stigma is the stigma perceived when having a painful inner struggle about a disability, even without any encounter with actual stigmatization [22]. Perception of stigma and experience of discrimination cause people to feel ashamed and may cause anxiety, depression, and isolation [37]. Measuring stigma is crucial because the evidence obtained from such assessment constitutes a valuable part of a situational analysis in the planning, monitoring, and evaluation of CBR service. Evidence obtained regarding intensity of stigma is helpful in advocating the participation rights of people with disabilities in society.

Evidence regarding measurement of stigma and participation is essential in building a strong evidence base for CBR in Chinese communities. Physical disabilities are regarded as visible disabilities and thus are immediately noticeable by an observer [33]. The effect of stigma on participation as experienced by people with physical disabilities is adverse, and it affects their mental health, physical health, and overall quality of life. In Chinese communities, lower self-concept and fewer quality social relationships are evident among people with physical disabilities as a result of stigmatization [4, 33]. For a comprehensive understanding and to assess participation and stigma among affected people, it is essential to have a validated instrument that can be effectively used by communities.

This study aimed to translate and validate two instruments, namely the Participation Scale and the Explanatory Model Interview Catalogue (EMIC) stigma scale, for use in Chinese communities. Both the Participation Scale and the EMIC focus on health-related stigma; the EMIC assesses perceived stigma and the Participation Scale assesses the impact of stigma on social participation [17]. These two scales are frequently used and put in the disability toolkit for use in community-based inclusive development programs [37]. The Participation Scale is an interview-based instrument for measuring the level of participation restriction of people with disabilities [36]. The instrument has good content validity because it covers most of the domains of participation in the International Classification of Functioning, Disability and Health [40]. Validation studies have demonstrated its high internal consistency (Cronbach’s alpha = 0.92), high interrater reliability (*r* = 0.80), and high discriminant validity for use with different target groups, such as people with leprosy and AIDS, in Nepal, India, and Brazil [36]. The EMIC stigma scale is an interview-based instrument for assessing perceived stigma. The EMIC stigma scale has been adopted in a non-Chinese context for people with HIV/AIDS and leprosy with acceptable discriminant and convergent validity, interitem reliability, and test–retest reliability [26, 30].

This study provides data for answering two research questions regarding the validity of the Participation Scale and EMIC stigma scale. First, the Participation Scale and the EMIC stigma scale are rarely employed to study people with physical disabilities. However, they are widely adopted in the fields of mental illness [25] and chronic disease [21]. It is unclear whether they can be equally valid when they are applied to people with physical disabilities. The second question is whether the validity of the Participation Scale and EMIC stigma scale in a Chinese cultural context is as clear as that in a non-Chinese context, where the stigmatization of disabilities in Chinese society is distinctively influenced by its traditional cultural values. The specific objectives of this study are:

1. To translate the Participation Scale and the EMIC stigma scale into a traditional Chinese version.
2. To examine the reliability and construct validity of the Participation Scale and the EMIC stigma scale.

## Methods

First, both the Participation Scale and the EMIC stigma scale were translated from English to Chinese according to the guidelines stated by the authors [17]. A back translation to English was performed by another bilingual translator. A panel of academic and clinical experts, including an occupational therapist, a clinical psychologist, and a sociologist, was formed to review the content validity of the Chinese version. Minor amendments to some of the wording were made to ensure readability. The psychometric properties and construct validity of the revised scales were examined.

### Participants

A total of 264 adults with physical disabilities were recruited for this study. People affiliated with the local organizations for persons with physical disabilities (DPOs) were targeted. Physical disabilities are operationally defined as a chronic physical impairment affecting one or more areas of the body, including the central nervous system, spinal cord, peripheral nervous system, and peripheral structures [8]. The inclusion criteria were (1) an age of 18 to 65 years; (2) not being in an acute phase of an illness or condition; (3) being mentally clear; and (4) having sufficient cognitive ability to comply with the instructions to complete the test. The participants were recruited from six types of DPO: ankylosing spondylitis, spinal cord injuries, developmental conditions with physical disabilities, brain damage, rheumatoid arthritis, and work-related orthopedic injuries.

DPOs were contacted and liaised by the principal investigator. Upon consent of the DPOs to participate in this study, the research team sent invitation letters and information sheets to all members. Ethical approval from the Committee on the Use of Human and Animal Subjects in Teaching and Research of Tung Wah College was obtained (HASC1415H04). All participants consented to participating in this study.

### Instruments

The Participation Scale is an 18-item interview-based instrument for measuring the level of participation among people with disabilities. When respondents reported restriction in a specific area (“no” or “sometimes”), they were asked to indicate the level of restriction. The choices were (1) no problem, (2) a small problem, (3) a moderate problem, and (4) a large problem. The sum of scores was calculated, with a higher total score representing a lower level of general participation. The respondents were ranked in five levels of participation by score: (1) no significant restriction (0–12), (2) mild restriction (13–22), (3) moderate restriction (23–32), (4) severe restriction (33–52), and (5) extreme restriction (53–90). The instrument, in its original language, has good content validity as it covers nine domains of participation: learning and applying knowledge, general tasks and demands, communication, mobility, self-care, domestic life, interpersonal interactions and relationships, major life areas, and community, social, and civil life. Van Brakel and colleagues [36] validated the instrument scores against expert scores and supported the external validity of the Participation Scale.

The EMIC stigma scale is a 15-item instrument, originally designed to measure stigma among leprosy-affected people. Because this study employed the EMIC stigma scale to measure stigma among people with physical disabilities, “leprosy” was replaced with “physical disability” in each question. Each question was measured with four options, which were “yes,” “possibly,” “uncertain,” and “no.” Scores were generated by assigning 3 points to “yes,” 2 to “possibly,” 1 to “uncertain,” and 0 to “no” for all questions except question 2, in which a reverse scoring method was employed. A composite score was obtained for each respondent by adding the scores of the 15 questions. A higher score implied a higher level of perceived stigma faced by the respondent. The internal consistency of the original scale (as applied in non-Chinese communities) is good, with a Cronbach’s alpha coefficient of 0.79.

### Data collection

Upon consent of the participants, the Participation Scale and the EMIC stigma scale were administered in a face-to-face interview. The interviewers were trained according to the guidelines and protocol of the IELP [17].

### Data analysis

Reliability analysis and convergent validity of the two instruments was performed using SPSS 21.0. Internal consistency and item-total correlation were examined. Reliability means that a measure consistently reflects the construct that it measures. Cronbach’s alpha was calculated to examine the internal consistency of the two scales. If a scale is reliable, the overall reliability is not expected to be greatly affected by any one item. It is therefore essential to also investigate the value of Cronbach’s alpha if an item is deleted. All values of alpha are approximately 0.8 or higher in a reliable scale. The values of the corrected item-total correlation should be above 0.3 to confirm that all items are correlated with the total score [11]. Convergent validity indicates that two measures that are considered to reflect the same underlying phenomenon will correlate significantly [28]. Convergent validity was tested by analyzing the correlation coefficient of the two measures. The Pearson product-moment correlation coefficient was planned if the data were found to be normally distributed; if not, the Spearman rank correlation coefficient was planned. This study used a score of *r* < 0.25 to indicate a weak correlation; *r* = 0.25 to 0.5 a moderate correlation; and *r* > 0.50 a strong correlation [11].

Exploratory factor analyses for the two scales were conducted separately using SPSS. Factor analysis entails examining the structure within numerous variables. Constructs must be defined by relevant measurable variables that can be collated to form a conceptual package called a factor. Using an exploratory approach to factor analysis allows the researcher to sort through numerous variables to reveal latent patterns of relationships among variables. When used to test the construct validity of an instrument, it simulates the process of theory testing, which means that the factors emerging from the process of analysis should match a hypothesized variable grouping [28]. The correlation of an individual item with a factor is called a factor loading. A correlation above +.30 or below −.30 indicates that an item contributes meaningfully to a factor [28]. In this study, exploratory factor analysis was performed using principal axis factoring with eigenvalues greater than 1. Principal axis factoring is preferable to principal component analysis because principal component analysis is only a data deduction method rather than factor analysis [7, 13]. The purpose of exploratory factor analysis is to derive a more parsimonious conceptual understanding of a set of variables by determining the number and nature of common factors required to account for the pattern of correlations among the measured constructs [10]. Exploratory factor analysis is based on the common-factor model. Principal axis factoring analyzes shared variance among the items. It is a factor analysis method that entails extracting factors on the basis of a reduced correlation matrix by using a priori communality estimates. Oblique rotation was used in this study because the factors might be correlated with each other. An oblique rotation theoretically renders a more accurate and reproducible solution, because it is generally expected in the social sciences that some correlation exists among factors [7]. The absolute values were suppressed in the coefficient display when the factor loading was less than 0.30.

Rasch model analyses for the EMIC stigma scale and the Participation Scale were conducted separately. The Rasch model is based on the concept that useful measurement involves examining only one human attribute at a time (unidimensionality) on a hierarchical line of inquiry [3]. If an instrument is valid, each of the items should contribute meaningfully to the construct being investigated, and the recorded performance is a reflection of a single underlying construct. A polytomous model was chosen in this study because both scales entail the use of a Likert scale to collect data and both had more than two response options. In this analysis, the data were first cleaned based on misfit person diagnosis. A person was excluded if the point measure correlation was negative, the outfit mean square value (MNSQ) was greater than 2, or the Z-standard value was greater than 2 [18]. Rasch model analysis was performed using Winsteps 3 software to examine the summary statistics, category structure, dimensionality, and model fit.

Dimensionality is a key part of the assessment of construct validity; it shows whether the items are measuring a single underlying dimension or several separate dimensions [12]. In Rasch model analysis, the principal component analysis of the residuals allows for a test of the local independence of items. The absence of any meaningful pattern in the residuals supports the assumption of unidimensionality. To confirm that the scale is unidimensional, the unexplained variance in the first contrast should not be greater than 2, and it should be smaller than the raw variance explained by the items [23]. Items were considered misfit if the point measure correlation was negative, the value of the ZSTD exceeded 2, and both the infit and outfit MNSQs exceeded 1.64 [1]. Notably, both exploratory factor analysis and Rasch model analysis were employed to test the dimensionality of the two scales. This study conducted exploratory factor analysis first and then Rasch model analysis. Exploratory factor analysis was conducted to explore the underlying concepts that the items are measuring, with the potential to explore the meaning of subscale scores. Rasch model analysis was performed to test a unidimensional score (measurement) scale. In this case, exploratory factor analysis revealed the patterns of relationships among items to form latent constructs [28]. It helped to define the underlying construct of participation (participation scale) and self-stigma (EMIC). Rasch model analysis entailed using data for measurement, and the objective of this analysis was to test and confirm a unidimensional interval scale [5]. In other words, exploratory factor analysis was used in this study as an exploratory device to make sense of the data. Once the factors had been identified, Rasch model analysis was used to further confirm that the measurement was unidimensional [31].

## Results

### Basic demographics

The Participation Scale and the EMIC stigma scale were administered to 264 adults with physical disabilities. All participants were aged 18 to 65; 50.8% were married; and 38.7% were of low socio-economic status. Types of condition were rheumatoid arthritis, acquired brain damage, spinal cord injury, ankylosing spondylitis, orthopedic injuries, and congenital physical disabilities. The basic demographics of the participants are shown in Table 1.

**Table 1:** Demographic and clinical characteristics of participants (*n* = 264)

Variables	Frequency	Percent
Gender		
Male	116	43.9
Female	148	56.1
Age		
18–25	8	3.0
26–35	21	8.0
36–45	44	16.7
46–55	85	32.2
56–65	106	40.1
Education		
Uneducated	2	0.8
Primary school	54	20.5
Secondary school	163	61.7
College	45	17.0
Marital status		
Single	80	30.3
Married	134	50.8
Divorced	32	12.1
Widowed	18	6.8
Household Income (HKD)		
< $5000	40	15.2
$5001 - $10,000	62	23.5
$10,001 - $ 20,000	79	30.0
$20,001 - $40,000	56	21.2
> $40,001	27	10.2
Condition		
Ankylosing spondylitis	31	11.7
Spinal cord injury	46	17.4
Congenital physical disabilities	18	6.8
Acquired brain damage	70	26.5
Rheumatoid arthritis	103	39.0
Orthopaedic injuries	27	10.2

### Reliability analysis

The Cronbach’s alpha values representing internal consistency were 0.93 and 0.897 for the Participation Scale and the EMIC stigma scale, respectively. If an item was deleted, the value of Cronbach’s alpha for all items in each scale was higher than 0.8 (Tables 2 and 3). The reliability of both the Participation Scale and the EMIC stigma scale was confirmed. However, the corrected item-total correlations for items 1 and 2 of the EMIC stigma scale were less than 0.3, meaning that they weakly correlated with the total score.

**Table 2:** Item-total Statistics of the Participation Scale

	Corrected Item-Total Correlation	Cronbach’s Alpha if Item Deleted
P-Scale		
1 Find job	.451	.930
2 Work as hard as others	.577	.927
3 Contribute to household economically	.568	.927
4 Travel outside your neighborhood	.722	.923
5 Take part in festivals	.729	.923
6 Take part in social activities	.715	.923
7 Socially active as peers	.739	.923
8 Have respect in the community	.602	.926
9 Have opportunity to take care of oneself and others	.582	.926
10 Have opportunity to enter into /maintain long term relationship	.570	.927
11 Visit other people in the community	.636	.925
12 Move around the house and village	.655	.925
13 Visit public places	.670	.924
14 Do household work	.589	.927
15 Opinion count in family discussion	.651	.925
16 Help other people	.687	.924
17 Comfortable meeting new people	.634	.925
18 Confident to learn and try new things	.586	.926

**Table 3:** Item-total Statistics of the EMIC stigma scale

	Corrected Item-Total Correlation	Cronbach’s Alpha if Item Deleted
EMIC		
1 Keep people from knowing about the disability	.188	.904
2 Discuss the problem with others	.222	.902
3 Reduce the pride or self-respect	.658	.887
4 Feel ashamed or embarrassed	.699	.885
5 Have less respect for you because of your problems	.693	.885
6 The contact might have bad effects on others	.657	.887
7 The others avoid you	.712	.885
8 Some people refuse to visit you	.675	.886
9 The colleagues and neighbors think less of your family	.664	.887
10 Cause problems for the children	.632	.888
11 Problem in getting married and marriage	.525	.893
12 makes it difficult for family members to get married	.632	.888
13 asked to stay away from social groups	.537	.892
14 Stay away from work or social groups	.551	.891
15 People think that you also have other health problems	.558	.891

### Convergent validity

The total scores of the two scales were correlated to test the convergent validity and assess the relationship of the two scales. Because the total scores of the two scales were not normally distributed, the Spearman’s rank order correlation was used in the analysis. The Spearman’s rank order correlation showed a moderate to strong correlation (*r* = 0.48, *p* = 0.001) among the findings of the two scales [11]. The convergent validity of the two scales was therefore confirmed.

### Exploratory factor analysis

Data were cleaned to exclude all cases with missing data from the analysis. For the exploratory factor analysis of the Participation Scale, a total of 256 valid cases were included. The Kaiser–Meyer–Olkin measure of sampling adequacy was 0.924, which meant that the data were adequate for exploratory factor analysis. Using principal axis factoring and oblique rotation (promax), three factors were extracted with eigenvalues greater than 1, and the absolute values of factor loadings less than 0.30 were suppressed (Table 4). Factor 1 comprised items 4 (travel outside your neighborhood), 5 (take part in festivals), 6 (take part in social activities), 7 (being as socially active as peers), 12 (move around the house and village), 13 (visit public places), and 14 (do household work). Factor 2 consisted of items 8 (have respect in the community), 9 (have opportunity to take care of oneself and others), 10 (have opportunity to enter into and maintain long-term relationships), 11 (visit other people in the community), 15 (opinion count in family discussion), 16 (help other people), 17 (comfortable meeting new people), and 18 (confident to learn and try new things). Factor 3 comprised items 1 (find job), 2 (work as hard as others), and 3 (contribute to household economically).

**Table 4:** Exploratory factor analysis of the Participation Scale (*n* = 256)

	Factor		
	1	2	3
4 Travel outside your neighborhood	.879		
13 Visit public places	.874		
12 Move around the house and village	.735		
7 Socially active as peers	.652		
6 Take part in social activities	.630		
5 Take part in festivals	.578		
14 Do household work	.444		
18 Confident to learn and try new things		.889	
17 Comfortable meeting new people		.887	
8 Have same respect in the community		.691	
16 Help other people		.454	
10 Have opportunity to enter into /maintain long term relationship		.428	
15 Opinion count in family discussion		.420	.331
9 Have opportunity to take care of oneself and others		.387	
11 Visit other people in the community	.336	.340	
1 Find job			.800
2 Work as hard as others			.751
3 Contribute to household economically			.555

Note. Absolute values were blanked in the coefficient display when the factor loading was less than 0.30

For the EMIC stigma scale, a total of 245 cases were included in the analysis. The value of the Kaiser–Meyer–Olkin measure of sampling adequacy was 0.905. Using principal axis factoring and oblique rotation (promax), two factors were extracted with eigenvalues of greater than 1, and the absolute values of factor loadings less than 0.30 were suppressed (Table 5). Factor 1 comprised items 3 (reduced pride or self-respect), 4 (feel ashamed or embarrassed), 5 (neighbors, colleagues, or others have less respect), 6 (contact might have bad effects on others), 7 (others avoid you), 8 (some people refuse to visit you), 9 (colleagues and neighbors think less of your family), 10 (cause problems for the children), 11 (problem in getting married and marriage), 12 (the disease makes it difficult for family members to marry), 13 (have been asked to stay away from social groups), 14 (decided to stay away from work or social groups), and 15 (people think that you also have other health problems). Factor 2 consisted of items 1 (keep people from knowing about the disability) and 2 (discussing the problem with others), as shown in Table 4. Items 1 and 2 were evidently clustered in one factor of self-disclosure.

**Table 5:** Exploratory factor analysis of the EMIC (*n* = 245)

	Factor	
	1	2
5 Have less respect for you because of your problems	.762	
7 The others avoid you	.760	
9 The colleagues and neighbors think less of your family	.740	
8 Some people refuse to visit you	.731	
4 Feel ashamed or embarrassed	.684	
12 makes it difficult for family members to get married	.672	
6 The contact might have bad effects on others	.672	
15 People think that you also have other health problems	.654	
10 Cause problems for the children	.648	
14 Stay away from work or social groups	.626	
3 Reduce the pride or self-respect	.608	
11 Problem in getting married and marriage	.588	
13 asked to stay away from social groups	.576	
1 Keep people from knowing about the disability		.681
2 Discuss the problem with others		.566

Notes. Absolute values were blanked in the coefficient display when the factor loading was less than 0.30

### Rasch model analysis

#### Participation scale

##### *Internal validity*

Using data cleaning (as described in the previous section), six persons were removed from the data file of the Participation Scale. Determined from the summary statistics of the Rasch analysis, the item reliability was 0.72 and the person reliability was 0.81. These statistics demonstrate the good reliability of the scale and high replicability of person ordering, indicating that the results would not vary if this sample were given another parallel set of items measuring the same construct [3]. The commonly accepted range for the mean-square (MNSQ) is 0.6 to 1.4 and − 2 to + 2 for the standardized value (ZSTD) [3]. The results of the analysis showed that the person fit was good, with the infit and outfit MNSQs being 1.05 and 1.03 and the ZSTDs being − 0.4 and − 0.5, respectively. The item fit was confirmed as good, with the infit and outfit MNSQs being 1.01 and 1.03 and the ZSTDs being − 0.1 and 0.0. Cronbach’s alpha was 0.93, and the value of item separation was 1.59, which indicated good reliability of the Participation Scale.

##### *Dimensionality*

The Rasch-residual-based principal component analysis (PCAR) showed that the unexplained variance explained by the first contrast was 2.2 (6.9%), and the raw variance explained by the items was 29.7%. However, examining the misfit order of all items revealed that items 1 and 3 were misfitted because the value of the ZSTD exceeded 2 and both the infit and outfit MNSQs exceeded 1.64. With an objective to test and confirm a unidimensional interval measurement scale using Rasch model analysis, items 1 and 3 were removed from the scale and dimensionality of the 16-item Chinese version of the Participation Scale was evaluated. This 16-item scale was then found to be unidimensional because the unexplained variance in the first contrast was reduced to 2.0 (6.8%) and the raw variance explained by items was 27.8%.

Dimensionality across the three extracted factors was analyzed using Rasch model analysis to test and confirm a unidimensional interval measurement scale. When the items were grouped according to the three factors as extracted from the exploratory factor analysis, they were found to be unidimensional. For Factor 1, the eigenvalue for unexplained variance in the first contrast was 1.8 (12.4%), and the raw variance explained by items was 27.2%. For Factor 2, the unexplained variance in the first contrast was 1.7 (10.8%), and the raw variance explained by items was 26.7%. For Factor 3, the unexplained variance in the first contrast was 1.7.

Integrating the findings from testing of dimensionality, it is concluded that the Participation Scale is a multi-dimensional scale. If a unidimensional interval scale is required for measurement purposes, it is suggested to use this 16-item scale. This 16-item scale was then used in this study for determining item difficulty and person hierarchy.

##### *Item hierarchy*

**Fig. 1:**
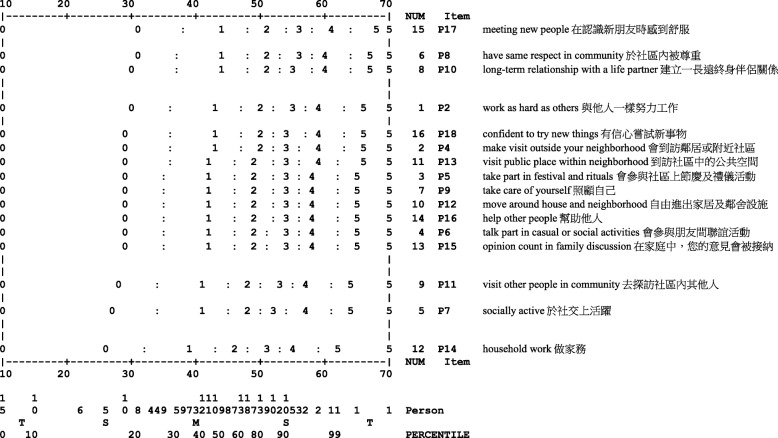
Item hierarchy of the 16-item Participation Scale as shown in the construct key map

The measurement scale must be unidimensional to determine the item difficulty and the ability of people. Accordingly, the item misfit order showed that items 1 and 3 were misfit. After these two items were removed from the scale, the 16-item scale was found to be unidimensional, and the examination of item difficulty and ability of people through Rasch model analysis was then legitimate. An item–person map was therefore computed based on the 16-item Chinese version Participation Scale. Rasch item maps traditionally show the distribution of item difficulties with the easiest items at the bottom and the most difficult items at the top. For the Participation Scale, the higher the score, the higher the participation restriction is. A successful outcome of CBR means a high level of participation. For judging enhanced participation, the interpretation of the results of the item–person map is reversed. In this case, the easiest items were at the top and the most difficult items were at the bottom. The item hierarchy was revealed clearly in the construct key map (Fig. 1). The least difficult items were items 17 (comfortable meeting new people), 8 (have respect in the community), and 10 (have opportunity to enter into and maintain long-term relationships). The most difficult items were items 14 (do household work), 7 (being as socially active as peers), and 11 (visit other people in the community).

##### *Scoring category structure*

The scoring structure of the Participation Scale was satisfactory because the average measures increased monotonically, which indicated that on average those with higher ability endorsed the higher category [3]. Table 6 shows that the average measures increased monotonically across the rating scales, which means that they functioned as expected. Moreover, fit statistics show that the outfit mean squares of every category was less than 2, meaning that no particular category introduced noise into the measurement process (Table 6).

**Table 6:** Category structure of the Participation Scale

Category label	Observed count	Observed count %	Observed average	Infit MNSQ	Outfit MNSQ	Threshold
0	1629	41	−15.06	1.25	1.14	None
1	891	22	−8.46	0.58	0.76	−6.60
2	652	16	−3.10	0.76	0.85	−2.14
3	490	12	0.44	0.81	0.87	0.97
4	1	0	0.97	0.97	0.69	62.56
5	337	8	3.40	1.04	1.35	−54.80

#### EMIC stigma scale

##### *Internal validity*

For the EMIC stigma scale, 245 persons were included in the analysis. The person reliability and item reliability were 0.74 and 0.90, respectively. The scale was found to be reliable. The person and item fit were both confirmed from the summary statistics. For person fit, the infit and outfit MNSQs were 1.09 and 1.11, whereas the infit and outfit ZSTDs were both − 0.1. For item fit, the infit and outfit MNSQs were 1.01 and 1.11, whereas the infit and outfit ZSTDs were − 0.3 and 0.3, respectively. The Cronbach’s alpha was 0.9, and the item separation was 3.04.

##### *Dimensionality*

The results of the principal component analysis showed that the eigenvalue of the unexplained variance in the first contrast was 1.9 (7.6%). The raw variance explained by items was 25.7%, which showed that the EMIC stigma scale was unidimensional.

##### *Item hierarchy*

**Fig. 2:**
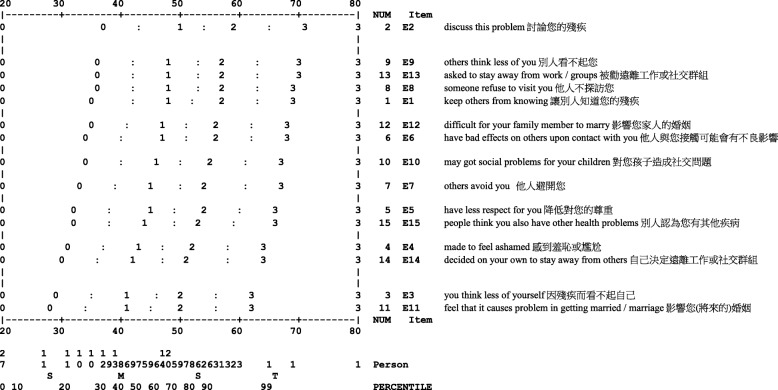
Item hierarchy of the EMIC stigma scale as shown in the construct key map

As for the Participation Scale, the interpretation of the item difficulty is reversed. The higher the score of the EMIC, the higher the level of perceived stigma. The most difficult item was Item 11 Problem in getting married and marriage. The easiest item was Item 2 Discuss the problem with others (Fig. 2).

##### *Scoring category structure*

The scoring structure of the EMIC stigma scale was satisfactory because the average measures of the four categories increased monotonically (Table 7). The fit statistics confirmed that the category function was good because the outfit MNSQs for all categories were less than 2.

**Table 7:** Category structure of the EMIC

Category label	Observed count	Observed count %	Observed average	Infit MNSQ	Outfit MNSQ	Threshold
0	1911	52	−13.09	0.97	1.01	None
1	754	21	−5.56	0.82	0.82	−1.56
2	524	14	−1.44	0.94	1.01	−0.15
3	484	13	3.08	1.10	1.45	1.70

## Discussion

### Participation scale

The results of the Rasch model analysis and the exploratory factor analysis were complementary, which helped provide a comprehensive perspective on the construct validity of the Participation Scale.

The results of the Rasch model analysis revealed that the Participation Scale is not unidimensional because the unexplained variance in the first contrast was greater than 2 in the PCAR. A previous study on the validation of the Participation Scale showed that the Participation Scale should be conceived as a two-factor model that consists of “work-related participation” (three items) and “general participation” (15 items) [32]. Our current study also indicates that work-related participation should be regarded as a distinct factor. Factor 3, which was extracted using exploratory factor analysis, consisted of all three items related to work and gainful employment.

With exploratory factor analysis, three factors were extracted from the Participation Scale. The extracted factors encompass the principal constructs related to disability and participation. The three factors can be described as (1) activity participation, (2) social engagement, and (3) work-related participation. Factor 1 is activity participation, which refers to the execution of physical and social activities [40], and is mainly performance-oriented participation [38]. Factor 2 is social engagement, which is togetherness-oriented participation [38] that focuses on performing meaningful social roles [14]. Factor 3 is work-related participation.

The item difficulties and abilities of people with physical disabilities were revealed by the Rasch model analysis. Item 14 (doing household work) was found to be the most difficult for people with physical disabilities. Items 7 (being as socially active as peers) and 11 (visiting others in the community) were ranked high in terms of difficulty for people with physical disabilities. These three items are related to physical activity (doing household work) and physical mobility (visiting others). This finding supports the construct validity of the scale because people with physical disabilities have different degrees of sensori-motor impairment that may limit their mobility and performance in physical activities [29]. Furthermore the individual experience of shame associated with physical disabilities may hinder a person from being as socially active as peers (Item 7). Shame is regarded as a strong emotion in Chinese culture. A person experiencing shame may feel that he or she has a stain that anyone around them can see [2]. Disability is associated with shame in Chinese communities, and the stigmatizing attitude is obvious [34]. Therefore, people with physical disabilities perceive that being as socially active as their peers is difficult.

### EMIC stigma scale

The Rasch model analysis and reliability analysis consistently showed that the EMIC stigma scale had a high internal consistency with a Cronbach’s alpha of 0.9. Generally, the internal consistency of an instrument is strong when its Cronbach’s alpha is higher than 0.70 [6]. The detailed statistics of the Rasch model analysis provided further corroborative support. The general principles of evaluating the Rasch model are to investigate outfit before infit, and to investigate the MNSQ before the ZSTD [23]. The outfit MNSQ was 1.11 for both person fit and item fit. The outfit MNSQ measures the size of the distortion of the outliers within the measurement system with expected values of 1 [23]. If the MNSQ remains between 0.5–1.5, it is evaluated as a productive measurement. The evaluation of the EMIC stigma scale and the Participation Scale as two distinct productive measurements was affirmed by the ZSTD outfit of person fit and item fit with an expected value of 0; the ZSTD aims to test a hypothesis of whether the data fit the model perfectly [23].

The reliability analysis should be studied in conjunction with the construct validity. The construct validity was examined with the aid of exploratory factor analysis with SPSS. The combined results of Rasch analysis and SPSS exploratory factor analysis showed that the EMIC stigma scale is a unidimensional measure of perceived self-stigma. The reason why Factor 1 stood apart from Factor 2 can be understood in the context of culture-bound syndromes, which stress the role played by culture in shaping the understanding of illness and health-related issues and places a heavy emphasis on the relativity of health and illness across cultures [35]. People with disabilities in Chinese society are particularly vulnerable to stigmatization because it is believed that they bring bad luck to the family and are being punished for immoral behaviors prior to their disability [20]. Among an adult sample in Hong Kong, those with visible disabilities scored significantly lower in self-concept than those without visible disabilities [33].

The EMIC evolved from Kleinman’s [19] pioneering work on an explanatory model of illness that not only embraces the integrity and complexity of cultural psychiatry and medical anthropology but also recognizes the necessity of incorporating an interaction between “emic” (an understanding of an illness within a cultural context) and “etic” (medical professionals’ understanding of an illness without a strict adherence to cultural beliefs), which was first introduced by Pike [27].

This study has limitations. A gold standard for measuring participation restriction and self-stigma has not been set. Both the EMIC stigma scale and the Participation Scale are feasible and robust in administration and measurement. This validation study confirms that the translated versions of both the EMIC stigma scale and the Participation Scale can effectively measure the level of participation and self-stigma for people with physical disabilities. However, the interrater and test-retest reliability have not been tested in this study. The generalizability of this study may be affected by the uneven age distribution of the participants. Furthermore, caution should be exercised when interpreting the total score of the Chinese version of the Participation Scale. The original version’s cutoff score could not be used here because the standards are based on other populations. This problem is compounded by the issue of the Chinese version’s dimensionality; therefore, further study is required if a norm or a cutoff score is required to differentiate grades of participation restriction.

## Conclusion

This study translated and validated the two scales for use with people with physical disabilities in Chinese communities. Results of the validation showed that both the Participation Scale and the EMIC stigma scale were valid and reliable. Although the interrater and test–retest reliability were not tested, this study sufficiently tested the internal reliability and construct validity of the Chinese Participation Scale and the EMIC stigma scale. The translated scales enable further development of the evidence-based practice of CBR because the effect of participation restriction and self-perceived stigma on people with disabilities can be accurately measured and documented.
